# Lactic Acid Production from Sugarcane Bagasse Hydrolysates by *Lactiplantibacillus* Strains

**DOI:** 10.1021/acsomega.5c08221

**Published:** 2025-10-26

**Authors:** Michelle C. A. Xavier, Giancarlo S. Dias, Saartje Hernalsteens, Telma T. Franco

**Affiliations:** 1 Federal University of Tocantins (UFT), NS-15 Ave., Quadra 109-Alcno 14, North, 77001-090 Palmas, Tocantins, Brazil; 2 State University of Campinas (UNICAMP), 500, Albert Einstein Ave., 13083-872 Campinas, São Paulo, Brazil; 3 Soochow University (SUDA), No. 1 Shizi Street, Suzhou, Jiangsu 215031, China

## Abstract

Lactic acid (LA) is a valuable organic acid widely used in many industries and bioplastics production. It can be produced by microbial fermentation of sugarcane bagasse (SCB), an abundant sugar industry waste rich in cellulose and hemicellulose. The aim of this study was to evaluate the ability of lactic acid bacteria to ferment pentoses from SCB hydrolysates for LA production in the presence or absence of hexoses. Initially, nine strains of *Lactiplantibacillus* spp*.* were screened for efficient xylose consumption. Subsequently, fermentation assays with the selected strain were performed in 2 L bioreactors using MRS-based media containing either hemicellulosic or total hydrolysates obtained from steam-exploded SCB. Among the nine strains, *Lactiplantibacillus pentosus* (*L. pentosus*) ATCC 8041 demonstrated superior xylose assimilation and LA production. Additionally, this strain was capable of consuming xylose in the presence of glucose, achieving an LA yield of 0.78 g/g and a maximum LA concentration of 28.99 g/L for MRS_TH_ media. The MRS_HEM_ was highly diluted, resulting in a maximum LA concentration and yield of 2.4 and 0.65 g/g, respectively. These results highlight the potential of *L. pentosus* ATCC 8041 to utilize both hemicellulose and cellulose fractions of SCB, offering promising avenues for sustainable lactic acid production from agroindustrial residues.

## Graphical abstract

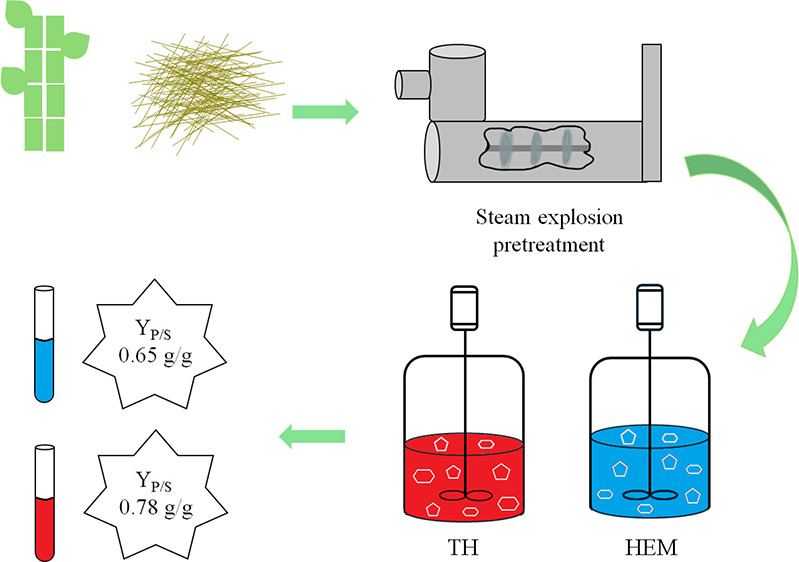

## Introduction

Lactic acid (2-hydroxy propionic acid) is an organic acid widely used in food, cosmetic, pharmaceutical, textile, and chemical industries.^1^ Recently, the increase in demand for biodegradable and bioabsorbable plastics, such as polylactic acid (PLA), has boosted global lactic acid (LA) production.^2^ LA serves as a building block for the synthesis of PLA plastic, a potential substitute of petroleum-based plastics, with several applications such as food packaging and surgical sutures.^3,4^ The global demand for LA is projected to reach 19.601 million tons by 2025, with an estimated market value of USD 9.8 billion.^5^

This organic acid can be produced through chemical synthesis or microbial fermentation. The microbial fermentation process yields optically pure l­(+) or d­(−)-LA, which are more valuable than the racemic DL form obtained via chemical synthesis.^6^ However, fermentative LA production is still largely dependent on glucose derived from refined sugar, a high-cost substrate. In this context, agricultural residues emerge as a viable alternative feedstock, offering sustainable, widely available, and low-cost sources of glucose.^7^ Such feedstocks not only reduce production costs but also align with environmental goals, contributing to the advancement of circular bioeconomy strategies.^8^

Agroindustrial residues contain glucose derived from cellulose as well as pentoses from hemicellulose, providing abundant additional fermentable sugars. However, most microorganisms cannot efficiently metabolize pentoses, limiting the valorization of this fraction.^8–11^ The microbial catabolism of pentoses is typically repressed by glucose, resulting in sequential sugar consumption. Once glucose is depleted, high concentrations of inhibitory end products hinder pentose assimilation, reducing LA yields and productivity.^12^ Therefore, screening microorganisms capable of fermenting both pentoses and hexoses is imperative to maximize LA production and reduce waste generation. Many microorganisms have been explored as potential producers of LA, including those belonging to the genera *Lactiplantibacillus*, *Aspergillus*, *Bacillus*, *Escherichia*, and *Saccharomyces*.^13^ Among them, lactic acid bacteria (LAB) are often utilized because of their superior safety, tolerance to acidic pH, and ability to achieve high LA yields and productivity.^5,14,15^ LAB can be classified as homofermentative, primarily utilizing glucose to obtain LA, or heterofermentative, producing LA, acetic acid, and ethanol.^16^

Sugarcane is one of the most widely cultivated crops worldwide, with Brazil and India as the leading producers.^17^ From its processing to extract juice for sugar and ethanol production, large amounts of bagasse are generated as a byproduct.^18^ Globally, annual sugarcane bagasse (SCB) production is estimated to surpass 48 million tons.^19^ Approximately 70% of the SCB generated is used as fuel in boilers and for cogeneration of energy in sugar plants, contributing to the sugar industry’s energy self-sufficiency.^20,21^ However, the remaining portion is typically stored in large, uncovered stockpiles, leading to environmental concerns such as dust emissions, groundwater contamination, spontaneous combustion, and the generation of contaminated leachates that can negatively affect the surrounding environment and nearby communities.^22^ To address these environmental issues, a promising strategy is to utilize the surplus SCB for LA production, given its high cellulose (32–45%) and hemicellulose (20–32%) contents.^23,24^

The strong covalent and hydrogen bonds within and between cellulose, hemicellulose, and lignin molecules require pretreatment strategies to efficiently release fermentable sugars.^25–27^ Steam explosion (SE) offers a low-energy and environmentally friendly approach to solubilize hemicellulosic sugars, disrupt lignocellulose fibers, and modify the cellulose structure. By increasing the accessibility of cellulose and hemicellulose, SE enhances the efficiency of enzymatic hydrolysis, leading to higher sugar yields.^28,29^ Compared to other technologies, SE offers improved scalability, consumes less energy, avoids recycling, and minimizes the formation of inhibitors like furfural, 5-hydroxymethylfurfural (HMF), and acetic acid that can inhibit LA production.^30^

The SCB hydrolysate, a relevant source of fermentable sugars, stands out as a low-cost substrate with high potential for the biotechnological production of LA. However, fermentative routes still face technical and economic challenges associated with lignocellulosic biomass processing. These include the presence of inhibitory compounds generated during pretreatment and saccharification, the efficient cofermentation of hexoses and pentoses by microorganisms, carbon catabolite repression, control of LA optical purity, and inhibition caused by substrate and end-product accumulation.^31,32^ In this context, the development of improved biomass pretreatment strategies, the selection and adaptation of microbial strains in real hydrolysates rich in second-generation (2G) sugars derived from SCB without detoxification steps, and the investigation of suitable fermentative process conditions are crucial to overcoming these barriers and, consequently, enhancing the industrial feasibility of LA production from lignocellulosic biomass.

Therefore, the present study aimed to obtain fermentable sugars from steam-exploded SCB and to investigate LA production from xylose by selecting the most efficient microorganism for the assimilation of this sugar and applying it in fermentative processes using hemicellulosic hydrolysate (HEM) and total hydrolysate (TH, containing cellulose and hemicellulose). In this way, the study seeks to meet the growing demand for LA while contributing to the development of an economically viable and environmentally sustainable bioprocess, in line with the principles of a circular bioeconomy, the valorization of agroindustrial residues, and the advancement of lignocellulosic biomass-based biorefineries.

## Methods

### Sugarcane Bagasse

The steam-exploded pretreated SCB was kindly provided by Usina Vale do Rosário (Orlândia, SP, Brazil). The material had an average moisture content of 58.52 ± 0.44% (w/w) and was stored in a freezer at −18 °C until use.

To obtain HEM, the pretreated bagasse was first soaked in water at a solid-to-liquid ratio of 1:10 (w/w), at 50 °C and 300 rpm (mechanical agitation) for 2 h. The solid fraction was then separated by using manual pressing. An additional volume of hot water (50 °C), equivalent to one-fifth of the initially added water, was subsequently added to the pressed cake, followed by a second manual pressing. The combined liquid fractions were centrifuged at 4500 rpm for 15 min and vacuum-filtered to remove insoluble lignin and residual solids. The HEM composition was 1.2 g/L glucose, 2.3 g/L xylose, 4.9 g/L acetic acid, and 0.6 g/L of arabinose.

The total hydrolysate (TH) was obtained by enzymatic hydrolysis performed simultaneously on both the HEM and the pressed cake, which was discarded during the hemicellulose extraction step. The enzymatic hydrolysis was conducted using a commercial enzyme preparation from Novozymes (Araucária, PR, Brazil). To minimize the presence of fine particles, the liquid fraction was filtered, adjusted to pH 4.8 with 25% (w/v) CaCO_3_, and reincorporated into the solid cake prior to enzymatic hydrolysis. Hydrolysis conditions followed the manufacturer’s recommendations regarding the enzyme dosage, pH, and temperature. The hydrolysis was carried out in agitated flasks at 50 °C and 300 rpm for 48 h using a mixture of 6% (v/v) cellulase–NS50013 (70 FPU/g), 0.6% (v/v) cellobiase–NS50010 (250 CBU/g), and 0.5% (v/v) xylanase–NS50030 (500 FXU/g). TH contained 21.7 g/L glucose, 13.9 g/L xylose, 7.4 g/L acetic acid, and 1.7 g/L arabinose.

### Microorganisms and Inoculum Preparation

Nine strains of the *Lactiplantibacillus* genus were used in this study, obtained from several banks of culture, including NRRL ARS Collection culture (Peoria, Illinois, USA), Fiocruz Foundation (Rio de Janeiro, RJ, Brazil), and André Tosello Foundation-CCT (Campinas, SP, Brazil).

The strains were reactivated in MRS medium containing glucose as the primary carbon source (MRS_glu_) and incubated at the optimum temperature for each microorganism, as shown in Table 1. The basal MRS medium contained (per liter) 10 g of soy peptone (Acumedia), 4.0 g of yeast extract (Himedia), 8.0 g of peptone A (peptic digest of an animal tissue) (Acumedia), 1 mL of Tween 80 (CAS 9005-65-6), 0.2 g of MgSO_4_·7H_2_O (CAS 10034-99-8), 0.05 g of MnSO_4_·H_2_O (CAS 10034-96-5), 5.0 g of sodium acetate (CAS 127-09-3), 2.0 g of K_2_HPO_4_ (CAS 7758-11-4), and 2.0 g of ammonium citrate tribasic (CAS 3458-72-8); th epH was adjusted to 6.0–6.5. The specific name of each medium reflected the carbon source used. For example, MRS_glu_ included 20 g/L glucose (CAS 50-99-7). All chemicals used were of analytical grade.

**Table 1:** Optimum Temperature for the Growth of Microorganisms

microorganisms	*T* (°C)
*Lactiplantibacillus pentosus* ATCC 8041	30
*Lactobacillus delbrueckii* NRRL B-445	37
*Lactobacillus delbrueckii* subsp*. delbrueckii* CCT 3744	37
*Lactobacillus delbrueckii* subsp*. lactis* ATCC 4797	37
*Lactobacillus delbrueckii* subsp*. lactis* ATCC 7830	37
*Lactiplantibacillus rhamnosus* ATCC 9595	37
*Lactiplantibacillus casei* subsp*.* *rhamnosus* NRRL B-442	37
*Lactiplantibacillus pentosus* NRRL B-227	28
*Lactococcus lactis* subsp*. lactis* NRRL B-4449	28

Strains were kept on MRS_glu_ agar slants at 4 °C until use. Inocula were prepared by transferring 1 mL of a preinoculum cell suspension into Erlenmeyer flasks containing 50 or 100 mL of culture medium, followed by incubation under the same conditions until the exponential growth phase was reached.

In addition to the glucose-based medium, other MRS-derived media were used with alternative carbon sources: xylose (MRS_xyl_), hemicellulosic hydrolysate (MRS_HEM_), and total hydrolysate (MRS_TH_).

### Screening Procedure

The *Lactiplantibacillus* strains were initially screened using an acidification assay on 9 cm Petri dishes (in duplicate) and inoculated by a single central stab. The plates contained MRS_xyl_ agar medium [MRS supplemented with 20 g/L xylose (CAS 58-86-6) and 20 g/L bacteriological agar (Himedia)] and adjusted to an initial pH of 6.5. A mixture of equal volumes of 0.5% aqueous bromocresol green and 0.5% aqueous bromocresol purple solutions was used as the pH indicator. Plates were incubated at each strain’s optimal growth temperature (Table 1), and color changes were evaluated after 24 h. Both the colony size and halo diameter were recorded, considering that the medium changes color from blue-violet (pH 6.2) to yellow (pH 4.2) in response to acidification.^33^

As a second screening step, strains that showed both growth and acidification on solid medium were selected for evaluation in a liquid MRS_xyl_ medium. Cultures were grown in 100 mL Erlenmeyer flasks containing 30 mL of medium, agitated at 150 rpm, and incubated at the optimum temperature for each strain (Table 1). *Lactococcus lactis* (*L. lactis*) subsp. *lactis* NRRL B-4449 and *Lactobacillus delbrueckii* (*L. delbrueckii*) NRRL B-445 exhibited poor cell development in MRS_xyl_. Therefore, alternative media proposed by Ohara et al.^34^ and Thomas^35^ were used for these strains, respectively, in an effort to improve growth performance.

### Lactic Acid Fermentation

Following strain selection, several assays were performed to evaluate the behavior of the selected strains in the presence of pentoses, from pure xylose to sugarcane bagasse-derived hydrolysates. All experiments were conducted in triplicate, and standard errors were below 10%.

The first assay was carried out in agitated Erlenmeyer flasks (100 mL) with a working volume of 30 mL and incubated at 30 °C and 150 rpm in MRS_xyl_ medium. The initial pH was adjusted to 6.0 prior to sterilization (121 °C, 15 min). Flasks were then inoculated with 10% (v/v) of the inoculum suspension. Samples were periodically collected to determine cell biomass concentration, total acidity (TA), and residual xylose concentration.

Fermentations using MRS_xyl_, MRS_HEM_, and MRS_TH_ media were performed in a 2 L Biostat A Plus bioreactor (Sartorius Stedim Biotech, Germany). The initial pH was adjusted to 6.0 using 4 M NaOH and/or 4 M H_3_PO_4_, followed by sterilization at 121 °C for 15 min. All fermentations were inoculated with a 10% (v/v) inoculum suspension. Bioreactor conditions were maintained at 30 °C, and the mixture was stirred at 150 rpm using two Rushton impellers. The pH was kept constant at 6.0 by automated addition of 20% (w/v) NaOH. The working volume was set to 1.1 L for both MRS_HEM_ and MRS_TH_ and 1.6 L for MRS_xyl_ fermentations. Samples were collected at various time points and centrifuged at 2395*g* for 10 min at room temperature, and the resulting supernatants were stored at −20 °C for subsequent analysis of glucose, xylose, arabinose, LA, and acetic acid concentrations.

### Analytical Methods

The microbial biomass concentration was indirectly estimated by measuring the optical density at 600 nm (OD_600_) and correlating the absorbance values with a standard curve established for each strain. Dry cell weight (DCW) was determined after centrifugation of the fermented broth at 2395*g* for 10 min, followed by a washing step with distilled water and a second centrifugation. The resulting biomass was dried at 60 °C for 24 h.

Glucose, xylose, arabinose, LA, and acetic acid concentrations were quantified using an ion chromatography system (Metrohm, Switzerland), consisting of a Professional IC 850 Anion-MCS-LP gradient pump, an 863 compact autosampler, an 871 Advanced Bioscan detector, and a 771 IC compact interface. For carbohydrate analysis, two Metrosep Carb 1–150 columns were connected in series. Separation was carried out at 30 °C using 100 mM NaOH as the mobile phase (flow rate: 1.0 mL/min), with an injection volume of 20 μL.^36,37^ For organic acid analysis, a Metrosep Organic Acid column (Metrohm AG CH 9101) was used, with 0.5 mM H_2_SO_4_ as the mobile phase and an injection volume of 196 μL.^38^

In the screening and flask-scale experiments, TA was determined via titration with 0.1 N NaOH, and the lactic acid content was calculated using eq 1.^39^

$$\mathrm{TA}\;(g/L)=\frac{\mathrm{NaOH}\;(\mathrm{mL})\times \mathrm{NaOH}\;\mathrm{normal}\times 90.08}{\mathrm{sample}\;(\mathrm{mL})}$$ 1

The concentration of d­(−)-LA was determined by using a commercial enzymatic kit (Boehringer Mannheim, Germany). The concentration of l­(+)-LA was calculated by subtracting the d-isomer concentration from the total LA content determined by ion chromatography.

Fermentation performance was evaluated by calculating product yield (*Y*_P/S_), biomass yield (*Y*_X/S_), and substrate conversion (conv.) using eqs 2–4, respectively. The maximum cell concentration (*X*_max_), maximum product productivity (*Q*_Pmax_), and maximum specific growth rate (μ′_max_) were derived from growth curve data.

$$Y_{P/S}\;(g/g)=(\frac{g\;\mathrm{lactic}\;\mathrm{or}\;\mathrm{acetic}\;\mathrm{acid}\;\mathrm{produced}}{g\;\mathrm{substrate}\;\mathrm{consumed}})$$ 2

$$Y_{X/S}\;(g/g)=(\frac{g\;\mathrm{cell}\;\mathrm{biomass}\;\mathrm{produced}}{g\;\mathrm{substrate}\;\mathrm{consumed}})$$ 3

$$\mathrm{conv}.\;(\%)=(\frac{\mathrm{initial}\;\mathrm{substrate}\;[g/L]-\mathrm{final}\;\mathrm{substrate}\;[g/L]}{\mathrm{initial}\;\mathrm{substrate}\;[g/L]})\times 100$$ 4

## Results and Discussion

### Screening of Strains Able to Assimilate and Ferment Xylose

Except for *L. delbrueckii* subsp. *lactis* ATCC 7830, all strains were able to utilize the medium components for growth and/or acid production, as shown in Figure S1 (in the Supporting Information). Although *L. pentosus* NRRL B-227 exhibited good colony growth, the surrounding halo remained blue-violet, indicating minimal acidification. Thomas^35^ reported that *L. delbrueckii* NRRL B-445 could assimilate xylose to produce LA in a medium containing only xylose. However, our screening assay failed to reproduce these findings. Notably, *L. pentosus* ATCC 8041 induced a halo color change earlier than the other strains, indicating a higher capacity for xylose assimilation and fermentation.

Since the initial results indicated that several strains were capable of metabolizing xylose as a carbon source, a second experiment was conducted in a liquid medium to obtain quantitative data to support strain selection. Table 2 summarizes the microbial biomass production, substrate consumption, and acid production observed during flask-scale fermentation.

**Table 2:** Kinetic Parameters of Xylose Fermentation in Agitated Flasks

microorganism	pH_End_	conv. (%)	TA (g/L)	*X*_max_ (g/L)	*Q*_Pmax_ (g/L/h)	*Y*_P/S_ (g/g)	*Y*_X/S_ (g/g)
*L. pentosus* ATCC 8041	3.8	92.2	17.88	1.75	0.39	0.85	0.13
*L. delbrueckii* NRRL B-445	5.5	25.3	0.75	0.27	0.04	0.17	0.09
*L. delbrueckii* subsp*. delbrueckii* CCT 3744	5.5	9.3	1.21	0.07	0.10	0.46	0.03
*L. delbrueckii* subsp*. lactis* ATCC 4797	5.5	19.8	1.14	0.24	0.01	0.27	0.05
*L. rhamnosus* ATCC 9595	5.7	31.1	0.82	0.64	0.01	0.11	0.17
*L. casei* subsp. *rhamnosus* NRRL B-442	5.4	28.7	1.18	0.57	0.11	0.20	0.08
*L. pentosus* NRRL B-227	5.5	17.0	1.41	0.79	0.01	0.43	0.19
*L. lactis* subsp*. lactis* NRRL B-4449	5.7	23.5	0.90	0.57	0.01	0.18	0.09
*L. delbrueckii* NRRL B-445	4.8	16.5	1.51	0.07	0.05	0.44	0.02

All strains were able to consume xylose, with conversion ranging from 9 to 92% (Table 2). *L. pentosus* ATCC 8041 stood out for its ability to utilize xylose as a carbon source for acid production, exhibiting the highest substrate consumption (92%), *Y*_P/S_ (0.85 g/g), and *Q*_Pmax_ (0.39 g/(L h)) after 30 h of cultivation. These findings, along with prior studies,^40–42^ confirm the suitability of *L. pentosus* ATCC 8041 for biotechnological applications involving pentose-rich hydrolysates, and therefore, this strain was selected for subsequent experiments.

### Lactic Acid Fermentation by *L. pentosus* ATCC 8041

*L. pentosus* ATCC 8041 exhibited the highest potential for converting xylose into acid. To further evaluate its performance, fermentation experiments were conducted in both shaker flasks and a bioreactor using an MRS_xyl_ medium. These assays aimed to validate the strain’s ability to utilize the carbon sources in MRS_xyl_ for cell growth and LA production.

Figure 1 shows the batch culture profiles of *L. pentosus* ATCC 8041 grown in MRS_xyl_ medium in shaker flasks. The growth curve showed no apparent lag phase, with immediate exponential growth and logarithmic production phase, up to 24 h of cultivation. After 48 h, the culture entered the deceleration phase, followed by growth stabilization. The biomass concentration reached a maximum of 1.75 g/L at 96 h. The total acidity, expressed as LA, peaked at 17.88 g/L after 120 h of fermentation, corresponding to an overall *Y*_P/S_ of 85.04%. The substrate conversion reached 92%, and the pH decreased from 6.0 to 3.8 within the first 72 h of fermentation.

**Figure 1:**
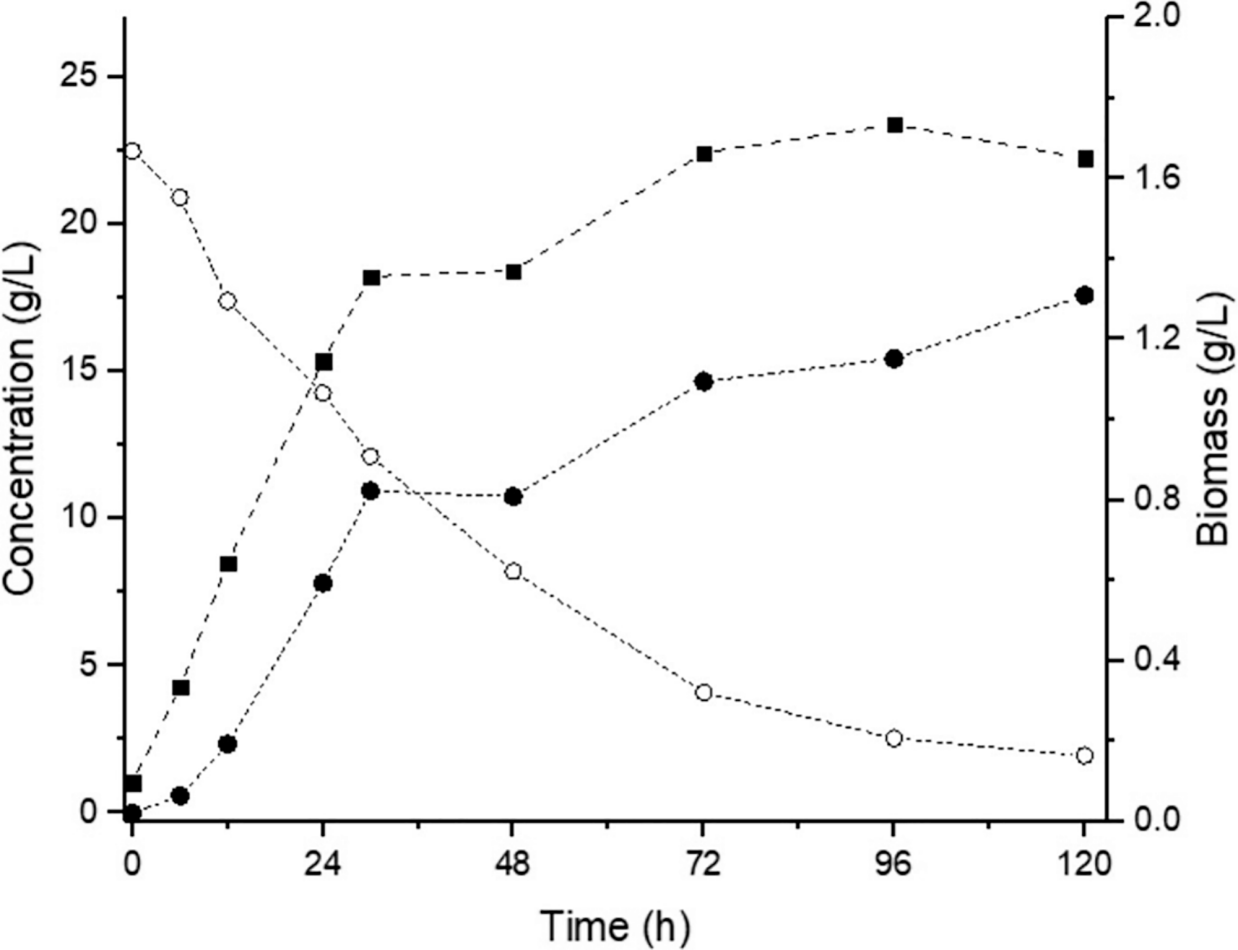
Growth profile, xylose consumption, and lactic acid production by *L. pentosus* ATCC 8041 for MRS_xyl_ medium. (Squares) Biomass, (open circles) xylose, and (solid circles) TA (total acidity expressed as LA).

In a 2 L bioreactor operated in simple batch mode with MRS_xyl_ medium (Figure 2), a lag phase for approximately 6 h was observed, followed by exponential growth. The biomass concentration reached 1.61 g/L, with a maximum specific growth rate (μ′_max_) of 0.157 h^–1^. Xylose was completely depleted after 48 h of fermentation, yielding 11.68 g/L d­(−)-LA and 13.06 g/L acetic acid. However, based on prior control experiments in MRS medium without addition of a carbohydrate source (data not shown), 4.91 g/L acetic acid was produced. Thus, it can be inferred that only 8.15 g/L of the total 13.06 g/L acetic acid produced in the MRS_xyl_ fermentation resulted from the fermentation of xylose, the sole carbohydrate source in this medium. These results highlight the ability of *L. pentosus* ATCC 8041 to convert xylose to organic acids efficiently.

**Figure 2:**
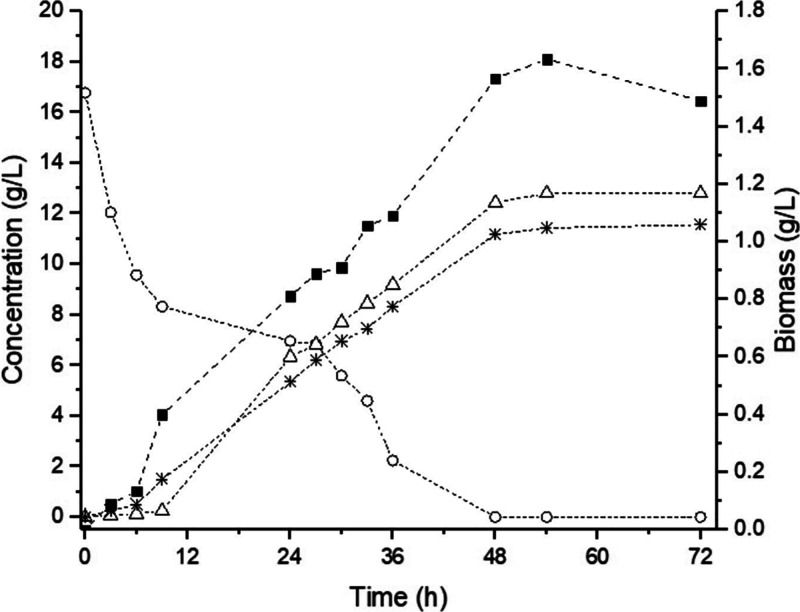
Profile of xylose consumption, cell growth, and LA and acetic acid production during batch fermentation (2 L bioreactor) of MRS_xyl_ medium by *L. pentosus* ATCC 8041. (Squares) Biomass, (circles) xylose, (asterisks) lactic acid, and (triangles) acetic acid.

The simultaneous xylose depletion and acetic acid formation indicate that *L. pentosus* ATCC 8041 metabolizes pentoses via the phosphoketolase pathway. In this mechanism, xylulose-5-phosphate is cleaved into glyceraldehyde-3-phosphate, which is further converted to LA, and acetyl-phosphate, which is metabolized to form acetic acid.^43^

Figure 3 illustrates the fermentation profile of *L. pentosus* ATCC 8041 in the MRS_HEM_ medium. The strain was able to simultaneously consume xylose, glucose, and arabinose (Figure 3a). Glucose was quickly depleted within the first 6 h of fermentation, while xylose and arabinose were metabolized at slower rates, indicating a preference for hexoses. Overall, 90% of the total sugar content was consumed, resulting in the production of 2.37 g/L LA (*Y*_P/S_ = 0.65 g/g), predominantly as the d­(−) isomer (66%), along with 0.99 g/L acetic acid (Figure 3b).

**Figure 3:**
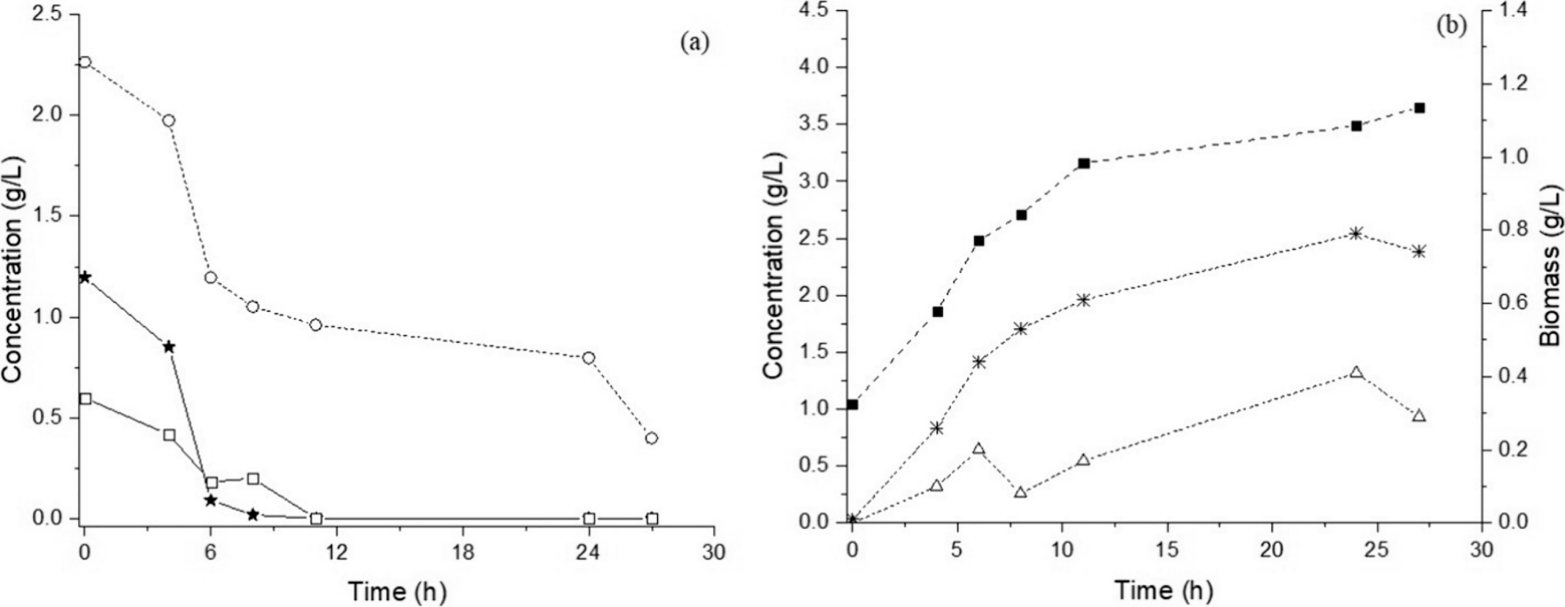
Fermentation profile of *L. pentosus* ATCC 8041 on MRS_HEM_ medium. (a) Profile of carbohydrate consumption and (b) cell growth and organic acid production. (Circles) Xylose, (stars) glucose, (open squares) arabinose, (solid squares) biomass, (asterisks) lactic acid, and (triangles) acetic acid.

The hemicellulosic fraction is often neglected in industrial bioprocesses, as lignocellulosic hydrolysis technologies have primarily targeted ethanol production. In this context, hemicellulose is not fermentable by non-GMO *Saccharomyces* strains and is typically treated as a waste or inhibitor-rich stream, containing compounds such as acetic acid, furfural, and HMF.^12^ However, the present study demonstrates the capacity of *L. pentosus* ATCC 8041 to effectively metabolize both hexoses and pentoses from the MRS_HEM_ medium.

The fermentation profile obtained with MRS_TH_ medium (Figure 4) was like that observed in the MRS_HEM_, with *L. pentosus* ATCC 8041 showing no evident lag phase and immediately entering exponential growth, suggesting an effective adaptation to the TH-based substrate. The microbial biomass reached 10.6 g/L after 20 h of fermentation, during which sugar depletion was observed. The μ′_max_ was attained after 2 h, reaching 0.17 h^–1^. Glucose was consumed first, followed by xylose and arabinose (Figure 4a). The LA concentration reached 28.99 g/L, with a *Y*_P/S_ of 0.78 g/g. The d­(−) isomer represented the predominant enantiomeric form (53%).

**Figure 4:**
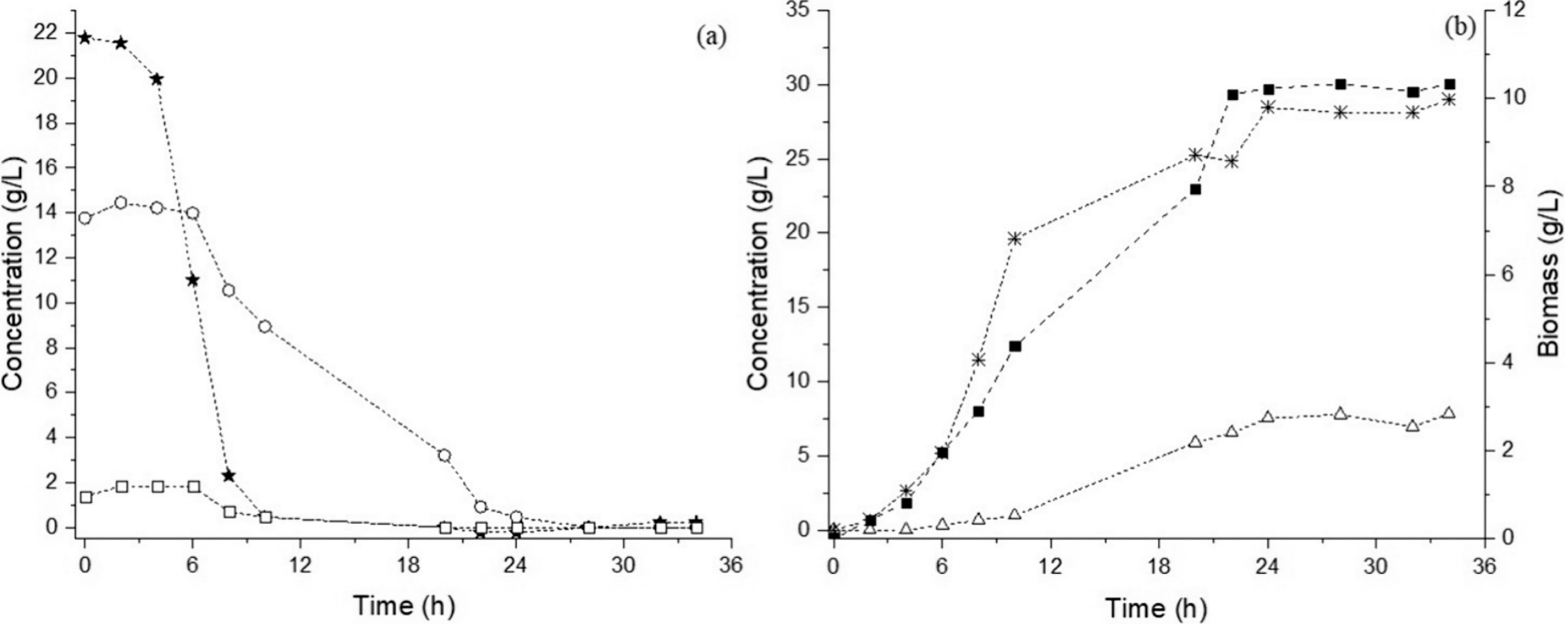
Fermentation profile of *L. pentosus* ATCC 8041 on MRS_TH_ medium. (a) Carbohydrate consumption and (b) cell growth and organic acids. (Circles) Xylose, (stars) glucose, (open squares) arabinose, (solid squares) biomass, (asterisks) lactic acid, and (triangles) acetic acid.

As shown in Figures 3 and 4, the microorganism preferentially consumes glucose, and then, the formation rate of acetic acid increases concomitantly with the consumption of xylose. These observations suggest that *L. pentosus* ATCC 8041 initially metabolizes glucose via the Embden–Meyerhof–Parnas pathway and subsequently switches to the phosphoketolase pathway for xylose fermentation, consistent with its facultative heterofermentative metabolism reported in previous studies.^44–46^ Similar behavior has been observed for other LAB, including *Lactiplantibacillus rhamnosus*, *Lactobacillus buchneri*, and *Lactococcus plantarum* (*L. plantarum*).^47–49^ The preference for glucose may be related to the higher ATP yield provided by the EMP compared to the PK pathway.^50^ It may be supported by the decrease in biomass formation observed immediately after glucose depletion.

Our findings demonstrate that *L. pentosus* ATCC 8041 efficiently fermented SCB hydrolysates without detoxification, resulting in high LA production. This performance likely results from its tolerance to inhibitors such as acetic acid, furfural, and HMF. Similarly, in a previous screening study, *L. pentosus* ATCC 8041 showed the highest LA concentration (16.2 g/L), yield (0.94 g/g), and productivity (0.34 g/L/h) among five LAB strains when cultivated on the SCB hydrolysate containing acetic acid (12.1 ± 2.5 g/L), furfural (0.19 ± 0.03 g/L), and HMF (0.06 ± 0.01 g/L).^51^ In contrast, other microorganisms like *Lactiplantibacillus casei* (*L. casei*) DSM 20011, *L. delbrueckii* DSM 20073, *L. lactis* DSM 20481, and *Bacillus smithii* DSM 4216 were inhibited by low concentrations of acetic acid, HMF, and furfural, as reported by van der Pol et al.^52^ Furthermore, strains such as *Bacillus coagulans* (*B. coagulans*) DSM 2314 ^53^ and *L. lactis* IO-1 ^54^ required detoxification steps with activated carbon or resins to ferment SCB hydrolysates.

*L. pentosus* ATCC 8041 has been successfully used for LA production from a wide variety of lignocellulosic substrates, consistently demonstrating a strong xylose utilization capacity. For example, Moldes et al.^55^ reported efficient fermentation of hydrolysates from trimming vine shoots, barley bran husks, corncobs, and *Eucalyptus globulus* chips, with the highest LA concentration obtained from barley bran husks (33 g/L), a substrate particularly rich in pentoses. Fermentation of grape marc also showed enhanced LA production (9.0 g/L after 12 h) compared to synthetic medium (6.0 g/L), likely due to its higher mineral and nitrogen content that favored xylose metabolism.^56^ In simultaneous saccharification and cofermentation processes, Acharjee et al.^15^ achieved 67 g/L LA from a 3:1 mixture of paper mill sludge and hemp hurd, while Zhu et al.^57^ obtained 74.8 g/L from corn stover in fed-batch mode. Recently, Yang et al.,^58^ using overlimed pretreated wood chip hydrolysates and a recombinant *L. pentosus* ATCC 8041 strain, scaled up a fermentation process from 5 to 9000 L to produce high-purity d-LA. In 5 L fermenters, the strain achieved a d-LA yield of 900.7 ± 141.4 mg/g with 99.8% optical purity. At 100 and 9000 L, the strain maintained excellent performance, exhibiting d-LA yields of 836.3 mg/g (95.0% OP) and 915.9 mg/g (93.8% OP), respectively. These studies reinforce the adaptability of *L. pentosus* ATCC 8041 not only to various pentose-rich hydrolysates but also to different fermentation strategies (batch, fed-batch, and large-scale processes). A summary of LA concentrations, yields, and substrate types for *L. pentosus* ATCC 8041 is provided in Table 3.

**Table 3:** Lactic Acid Production by *L. pentosus* ATCC 8041 from Agroindustrial Residues

substrate	substrate concentration (g/L)	*Y*_P/S_ (g/g)	lactic acid concentration (g/L)	references
sugarcane bagasse MRS_TH_	37.2	0.78	28.9	our work
sugarcane bagasse MRS_HEM_	4.1	0.65	2.4	our work
sugarcane bagasse (hemicellulose)	n.a.^a^	0.71	42.4	^51^
sugarcane bagasse (hemicellulose)	32.8	0.81	30.2	^59^
wheat straw	n.a.^a^	0.55	12.6	^41^
wheat straw	n.a.^a^	0.81	13.5	^46^
paper mill sludge and hemp hurd	n.a.^a^	0.60	22.4	^15^
brewery spent grain	n.a.^a^	0.65	8.94	^63^
chestnut burrs	n.a.^a^	0.92	6.56	^40^
trimming vine shoots	35.0	0.76	26.5	^55^
trimming vine shoots	33.4	0.74	24.5	^42^
grape marc	12.3	0.55	6.5	^56^
grape marc	12.5	0.71	7.2	^64^
corn stover	n.a.^a^	0.65	74.8	^57^
corn stover	50.0	0.38	8.31	^65^
waste eucalyptus globulus detoxicated	18.0	0.70	14.5	^55^
barley bran husks	57.0	0.57	33.7	^55^
corncobs	35.0	0.53	24.7	^55^
vine trimming	33.8	0.61	21.8	^66^
vine trimming	27.8	0.77	21.8	^67^
wood chips	74.6	0.85	63.2	^58^

a Not available in the original reference.

However, despite this broad applicability, studies specifically addressing the fermentation of SCB hydrolysates for LA production using *L. pentosus* ATCC 8041 remain limited. González-Leos et al.^59^ used two-stage acid treatment to obtain HEM hydrolysates from SCB. The HEM hydrolysate was supplemented with 10 g/L yeast extract and 10 g/L sterilized corn steep liquor and then fermented, obtaining an LA concentration of 30.178 g/L, a productivity of 0.369 g/L/h, and a *Y*_P/S_ of 0.811 g/g. Wischral et al.,^51^ after alkaline-acid pretreatment, fermented the SCB HEM hydrolysate to produce 16.2 g/L LA, with a *Y*_P/S_ of 0.94 g/g and a productivity of 0.34 g/L/h. They subsequently applied a saccharification and cofermentation strategy, in which partially delignified cellulignin, an enzyme cocktail, the HEM hydrolysate, and *L. pentosus* ATCC 8041 were simultaneously introduced into the bioreactor to produce LA. This approach achieved 65 g/L LA with a yield of 0.93 g/g and a productivity of 1.01 g/L/h. In contrast, other laboratories have been extensively investigated. For example, *L. casei* TISTR 390 exhibited an LA concentration of 21.3 g/L after 120 h with a maximum productivity of 0.63 g/L/h.^60^ *B. coagulans* DSM ID 14-300 produced 56 g/L LA in 49 h from supplemented SCB hydrolysates. The bacterium also detoxified the medium by metabolizing HMF and furfural, although productivity was decreased due to a prolonged adaptation phase.^61^ *L. lactis* TISTR 1401, *L. delbrueckii* TISTR 326, *L. plantarum* TISTR 543, or *L. casei* TISTR 390 produced approximately 11 g/L LA after 96 h of fed-batch fermentation. The relatively low LA concentration observed for all strains was attributed to the inhibition effects of acidity.^62^

Overall, SCB offers a promising, low-cost, and renewable agricultural residue for LA production (Table 3). Our results also demonstrate that SE pretreatment can be effectively applied to SCB, offering a scalable alternative to the acid pretreatment commonly reported in the literature. Compared with other strains, *L. pentosus* ATCC 8041 achieved high LA production without requiring additional detoxification or supplementation, highlighting its potential for industrial applications.

## Conclusions

*L. pentosus* ATCC 8041 demonstrated superior xylose assimilation and LA production capabilities among the nine *Lactiplantibacillus* strains evaluated in both solid and liquid MRS_xyl_ media. Moreover, this strain efficiently consumed xylose to produce LA in both shaker flasks and a 2 L bioreactor, although a lag phase was observed during scale-up. SE proved to be an effective pretreatment for recovering fermentable sugars from SCB. The resulting HEM was highly diluted, and further concentration steps, such as evaporation, could be employed to increase the hexose and pentose levels. *L. pentosus* ATCC 8041 successfully cofermented glucose, xylose, and arabinose from both hemicellulosic and total hydrolysates, achieving in MRS_TH_ medium a high LA concentration of 28.99 g/L with a yield of 0.78 g/g. The fermentation profile revealed a metabolic shift from homo- to heterofermentation upon glucose depletion, as evidenced by acetic acid coproduction. Overall, these findings highlight the potential of *L. pentosus* ATCC 8041 for valorizing sugarcane bagasse hydrolysates without detoxification steps. This contributes to the sustainable use of agricultural residues and advances circular biotechnology by enabling the bioconversion of residual lignocellulosic biomass into value-added products.

## Supplementary Material



## Notes

Images of acidification tests in solid MRS_xyl_ medium for *Lactobacillus* strains (PDF)

## References

[1] Meruvu H., Harsa S. T. (2023). Lactic Acid Bacteria: Isolation–Characterization
Approaches and Industrial Applications. Crit.
Rev. Food Sci. Nutr..

[2] Wu Y., Gao X., Wu J., Zhou T., Nguyen T. T., Wang Y. (2023). Biodegradable
Polylactic Acid and Its Composites: Characteristics, Processing, and
Sustainable Applications in Sports. Polymers..

[3] Reshma C. S., Remya S., Bindu J. (2024). A Review of
Exploring the Synthesis,
Properties, and Diverse Applications of Poly Lactic Acid with a Focus
on Food Packaging Application. Int. J. Biol.
Macromol..

[4] Maruichi Y., Nomura T., Minami E., Kawamoto H. (2025). Lactic Acid Fermentation
of Sugars Produced by Fast Pyrolysis of Cellulose and Effects of By-Products
on Fermentation. Bioresour. Technol. Reports.

[5] Huang Y., Wang Y., Shang N., Li P. (2023). Microbial Fermentation
Processes of Lactic Acid: Challenges, Solutions, and Future Prospects. Foods.

[6] Song L., Yang D., Liu R., Liu S., Dai L., Dai X. (2022). Microbial Production of Lactic Acid
from Food Waste: Latest Advances,
Limits, and Perspectives. Bioresour. Technol..

[7] Nwamba M. C., Sun F., Mukasekuru M. R., Song G., Harindintwali J. D., Boyi S. A., Sun H. (2021). Trends and
Hassles in the Microbial
Production of Lactic Acid from Lignocellulosic Biomass. Environ. Technol. Innov..

[8] Augustiniene E., Jonuskiene I., Kailiuviene J., Mazoniene E., Baltakys K., Malys N. (2023). Application
of Whole-Cell Biosensors
for Analysis and Improvement of L- and D-Lactic Acid Fermentation
by *Lactobacillus Spp*. from the Waste of Glucose Syrup
Production. Microb. Cell Fact..

[9] Gunkova P. I., Buchilina A. S., Maksimiuk N. N., Bazarnova Y. G., Girel K. S. (2021). Carbohydrate Fermentation
Test of Lactic Acid Starter
Cultures. IOP Conf. Ser.: Earth Environ. Sci..

[10] Alexandri M., Hübner D., Schneider R., Fröhling A., Venus J. (2022). Towards Efficient Production of Highly Optically Pure D-Lactic Acid
from Lignocellulosic Hydrolysates Using Newly Isolated Lactic Acid
Bacteria. N. Biotechnol..

[11] Reis
Kemita L., França Lopes da Silva L., Pratto B. (2024). Optimizing Dilute Acid Pretreatment for Enhanced Recovery
and Co-Fermentation of Hexose and Pentose Sugars for Ethanol and Butanol
Production. Fuel.

[12] Kim J., Block D. E., Mills D. A. (2010). Simultaneous Consumption of Pentose and
Hexose Sugars: An Optimal Microbial Phenotype for Efficient Fermentation
of Lignocellulosic Biomass..

[13] Sionek B., Szydłowska A., Küçükgöz K., Kołożyn-Krajewska D. (2023). Traditional and New Microorganisms
in Lactic Acid Fermentation of Food. Fermentation.

[14] Sionek B., Szydłowska A., Trząskowska M., Kołożyn-Krajewska D. (2024). The Impact
of Physicochemical Conditions on Lactic Acid Bacteria Survival in
Food Products. Fermentation.

[15] Acharjee T., Guan W., Lee Y. N., Jiang Z. (2018). Production
of Lactic
Acid from Mixed Feed of Paper Mill Sludge and Hemp Hurd by Simultaneous
Saccharification and Co-Fermentation. Sci. Technol.
For. Prod. Process.

[16] You S., Jia Y., Ge G., Du S. (2025). Revealing the Underlying Potential
Mechanisms of Lactic Acid Bacteria-Mediated Anaerobic Fermentation
of Native Grass by Microbiome and Metagenomic. J. Agric. Food Res..

[17] Zachariah J. P., Jakka R. S. (2025). Experimental Investigation on Shear
Behavior and Mechanical
Properties of Fine Sand Reinforced with Sugarcane Bagasse Fibers. Int. J. Geosynth. Gr. Eng..

[18] Vandenberghe L. P. S., Valladares-Diestra K. K., Bittencourt G. A., Zevallos Torres L. A., Vieira S., Karp S. G., Sydney E. B., de Carvalho J. C., Thomaz Soccol V., Soccol C. R. (2022). Beyond Sugar and
Ethanol: The Future of Sugarcane Biorefineries in Brazil. Renew. Sustain. Energy Rev..

[19] Khan M. A., Zhang B., Ahmad M., Niekurzak M., Khan M. S., Sabri Sabri M. M., Chen W. (2025). Optimizing Concrete
Sustainability with Bagasse Ash and Stone Dust and Its Impact on Mechanical
Properties and Durability. Sci. Rep..

[20] Meghana M., Shastri Y. (2020). Sustainable Valorization
of Sugar Industry Waste: Status,
Opportunities, and Challenges. Bioresour. Technol..

[21] Hiranobe C. T., Gomes A. S., Paiva F. F. G., Tolosa G. R., Paim L. L., Dognani G., Cardim G. P., Cardim H. P., dos Santos R. J., Cabrera F. C. (2024). Sugarcane Bagasse:
Challenges and Opportunities for
Waste Recycling. Clean Technol..

[22] Rainey T. J., O’Hara I. M., Mann A. P., Bakir C. H., Plaza F. (2013). Effect of
Depithing on the Safety and Environmental Aspects of Bagasse Stockpiling. Process Saf. Environ. Prot..

[23] Alokika, Anu, Kumar A., Kumar V., Singh B. (2021). Cellulosic
and Hemicellulosic Fractions of Sugarcane Bagasse: Potential,
Challenges and Future Perspective. Int. J. Biol.
Macromol..

[24] Barciela P., Perez-Vazquez A., Fraga-Corral M., Prieto M. A. (2023). Utility Aspects
of Sugarcane Bagasse as a Feedstock for Bioethanol Production: Leading
Role of Steam Explosion as a Pretreatment Technique. Processes.

[25] Baksi S., Saha D., Saha S., Sarkar U., Basu D., Kuniyal J. C. (2023). Pre-Treatment of Lignocellulosic Biomass: Review of
Various Physico-Chemical and Biological Methods Influencing the Extent
of Biomass Depolymerization. Int. J. Environ.
Sci. Technol..

[26] Azad S. A., Madadi M., Rahman A., Sun C., Sun F. (2025). Machine Learning-Driven
Optimization of Pretreatment and Enzymatic Hydrolysis of Sugarcane
Bagasse: Analytical Insights for Industrial Scale-Up. Fuel.

[27] Guilherme A. D. A., Dantas P. V. F., Soares J. C. J., Santos E. S. D., Fernandes F. A. N., Macedo G. R. D. (2017). Pretreatments
and Enzymatic Hydrolysis of Sugarcane
Bagasse Aiming at the Enhancement of the Yield of Glucose and Xylose. Brazilian J. Chem. Eng..

[28] Geng C., Liu S. Q., Lu Y. (2025). Enzymatic
Hydrolysis Combined with
Probiotic Lactic Acid Bacteria Fermentation Enhances the Nutritional
Value and Flavor Profile of Kombu (*Saccharina Japonica*) Slurry. Food Biosci..

[29] Baksi S., Sarkar U., Villa R., Basu D., Sengupta D. (2023). Conversion
of Biomass to Biofuels through Sugar Platform: A Review of Enzymatic
Hydrolysis Highlighting the Trade-off between Product and Substrate
Inhibitions. Sustain. Energy Technol. Assessments.

[30] Duque, A. ; Manzanares, P. ; Ballesteros, I. ; Ballesteros, M. Steam Explosion as Lignocellulosic Biomass Pretreatment. In Biomass Fractionation Technologies for a Lignocellulosic Feedstock Based Biorefinery; Mussato, S. I. , Ed.: Elsevier, 2016, 349–368. 10.1016/B978-0-12-802323-5.00015-3.

[31] Agrawal D., Kumar V. (2023). Recent Progress on Sugarcane-Bagasse
Based Lactic Acid Production:
Technical Advancements. Potential and Limitations.
Ind. Crops Prod..

[32] Haokok C., Lunprom S., Reungsang A., Salakkam A. (2023). Efficient Production
of Lactic Acid from Cellulose and Xylan in Sugarcane Bagasse by Newly
Isolated *Lactiplantibacillus Plantarum* and *Levilactobacillus Brevis* through Simultaneous Saccharification
and Co-Fermentation Process. Heliyon.

[33] Venus J., Idler F., Albrecht C. (1992). New Ways of
Selecting Lactic Acid
Bacteria for Biotechnological Processes. Appl.
Microbiol. Biotechnol..

[34] Ohara H., Owaki M., Sonomoto K. (2007). Calculation
of Metabolic Flow of
Xylose in *Lactococcus lactis*. J. Biosci. Bioeng..

[35] Thomas S. (2000). Production
of Lactic Acid from Pulp Mill Solid Waste and Xylose Using *Lactobacillus delbrueckii* (NRRL B445). Appl. Biochem. Biotechnol. - Part A Enzym. Eng. Biotechnol..

[36] Walford S. N. (2002). Applications
of Ion Chromatography in Cane Sugar Research and Process Problems. J. Chromatogr. A.

[37] Tapia
V E., Anschau A., Coradini A. L., T Franco T., Deckmann A. C. (2012). Optimization
of Lipid Production by the Oleaginous Yeast Lipomyces Starkeyi by
Random Mutagenesis Coupled to Cerulenin Screening. AMB Express.

[38] Anschau, A. Lipid Production by Lipomyces Starkeyi: Strategy to Obtain High Cell Density from Xylose and Glucose; Ph.D. Thesis; University of Campinas (UNICAMP): Campinas, Brazil, 2014.

[39] Feng S., Xiang S., Bian X., Li G. (2020). Quantitative Analysis
of Total Acidity in Aqueous Lactic Acid Solutions by Direct Potentiometric
Titration. Microchem. J..

[40] Costa-Trigo I., Otero-Penedo P., Outeiriño D., Paz A., Domínguez J. M. (2019). Valorization
of Chestnut (*Castanea Sativa*) Residues: Characterization
of Different Materials and Optimization of the Acid-Hydrolysis of
Chestnut Burrs for the Elaboration of Culture Broths. Waste Manag..

[41] Cubas-Cano E., González-Fernández C., Ballesteros M., Tomás-Pejó E. (2019). *Lactobacillus
Pentosus* CECT 4023 T Co-Utilizes Glucose and Xylose to Produce
Lactic Acid
from Wheat Straw Hydrolysate: Anaerobiosis as a Key Factor. Biotechnol. Prog..

[42] Bustos G., de la Torre N., Moldes A. B., Cruz J. M., Domínguez J. M. (2007). Revalorization
of Hemicellulosic Trimming Vine Shoots Hydrolyzates Trough Continuous
Production of Lactic Acid and Biosurfactants by *L*. Pentosus. J. Food Eng..

[43] Abdel-Rahman M. A., Tashiro Y., Sonomoto K. (2011). Lactic Acid
Production from Lignocellulose-Derived
Sugars Using Lactic Acid Bacteria: Overview and Limits. J. Biotechnol..

[44] Narisetty V., Cox R., Bommareddy R., Agrawal D., Ahmad E., Pant K. K., Chandel A. K., Bhatia S. K., Kumar D., Binod P., Gupta V. K., Kumar V. (2021). Valorisation of Xylose to Renewable
Fuels and Chemicals, an Essential Step in Augmenting the Commercial
Viability of Lignocellulosic Biorefineries. Sustain. Energy Fuels.

[45] Cubas-Cano E., González-Fernández C., Ballesteros I., Tomás-Pejó E. (2020). Efficient Utilization
of Hydrolysates
from Steam-Exploded Gardening Residues for Lactic Acid Production
by Optimization of Enzyme Addition and PH Control. Waste Manag..

[46] Cubas-Cano E., González-Fernández C., Tomás-Pejó E. (2019). Evolutionary
Engineering of *Lactobacillus Pentosus* Improves Lactic
Acid Productivity from Xylose-Rich Media at Low PH. Bioresour. Technol..

[47] Klongklaew A., Unban K., Kalaimurugan D., Kanpiengjai A., Azaizeh H., Schroedter L., Schneider R., Venus J., Khanongnuch C. (2023). Bioconversion
of Dilute Acid Pretreated
Corn Stover to L-Lactic Acid Using Co-Culture of Furfural Tolerant *Enterococcus Mundtii* WX1 and *Lactobacillus Rhamnosus* SCJ9. Fermentation.

[48] Liu S., Skinner-Nemec K. A., Leathers T. D. (2008). *Lactobacillus Buchneri* Strain NRRL
B-30929 Converts a Concentrated Mixture of Xylose and
Glucose into Ethanol and Other Products. J.
Ind. Microbiol. Biotechnol..

[49] Zhang Y., Vadlani P. V. (2015). Lactic Acid Production
from Biomass-Derived Sugars
via Co-Fermentation of *Lactobacillus Brevis* and *Lactobacillus Plantarum*. J. Biosci.
Bioeng..

[50] Årsköld E., Lohmeier-Vogel E., Cao R., Roos S., Rådström P., Van Niel E. W. J. (2008). Phosphoketolase Pathway Dominates
in *Lactobacillus
Reuteri* ATCC 55730 Containing Dual Pathways for Glycolysis. J. Bacteriol..

[51] Wischral D., Arias J. M., Modesto L. F., de França Passos D., Pereira N. (2019). Lactic Acid Production
from Sugarcane Bagasse Hydrolysates
by *Lactobacillus Pentosus*: Integrating Xylose and
Glucose Fermentation. Biotechnol. Prog..

[52] van
der Pol E. C., Vaessen E., Weusthuis R. A., Eggink G. (2016). Identifying Inhibitory Effects of Lignocellulosic By-Products
on Growth of Lactic Acid Producing Micro-Organisms Using a Rapid Small-Scale
Screening Method. Bioresour. Technol..

[53] Alves W. R., da Silva T. A., Zandoná
Filho A., Pereira
Ramos L. (2023). Lactic Acid Production from Steam-Exploded Sugarcane Bagasse Using *Bacillus Coagulans* DSM2314. Fermentation.

[54] Laopaiboon P., Thani A., Leelavatcharamas V., Laopaiboon L. (2010). Acid Hydrolysis
of Sugarcane Bagasse for Lactic Acid Production. Bioresour. Technol..

[55] Moldes A. B., Torrado A., Converti A., Domínguez J. M. (2006). Complete
Bioconversion of Hemicellulosic Sugars from Agricultural Residues
into Lactic Acid by *Lactobacillus Pentosus*. Appl. Biochem. Biotechnol..

[56] Rivera Ó. M. P., Torrado A. M., Moldes A. B., Domínguez J. M. (2009). Minerals
and Organic Nitrogen Present in Grape Marc Hydrolyzates Enhance Xylose
Consumption by *Lactobacillus Pentosus*. Appl. Biochem. Biotechnol..

[57] Zhu Y., Lee Y. Y., Elander R. T. (2007). Conversion
of Aqueous Ammonia-Treated
Corn Stover to Lactic Acid by Simultaneous Saccharification and Cofermentation. Appl. Biochem. Biotechnol..

[58] Yang C. C., Chou C. H., Guo G. L., Lin Y. J., Wen F. S. (2024). From Agricultural
Biomass to D Form Lactic Acid in Ton Scale via Strain Engineering
of *Lactiplantibacillus pentosus*. Bioresour. Technol..

[59] González-Leos A., Bustos-Vázquez M. G., Rodríguez-Castillejos G. C., Rodríguez-Durán L. V., Del Ángel-Del Ángel A. (2019). Kinetics of
lactic acid fermentation from sugarcane bagasse by *Lactobacillus
Pentosus*. Rev. Mex. Ing. Química.

[60] Oonkhanond B., Jonglertjunya W., Srimarut N., Bunpachart P., Tantinukul S., Nasongkla N., Sakdaronnarong C. (2017). Lactic Acid
Production from Sugarcane Bagasse by an Integrated System of Lignocellulose
Fractionation, Saccharification, Fermentation, and Ex-Situ Nanofiltration. J. Environ. Chem. Eng..

[61] Alves
de Oliveira R., Schneider R., Vaz Rossell C. E., Maciel Filho R., Venus J. (2019). Polymer Grade L-Lactic Acid Production
from Sugarcane Bagasse Hemicellulosic Hydrolysate Using *Bacillus
Coagulans*. Bioresour. Technol. Reports.

[62] Jonglertjunya W., Pranrawang N., Phookongka N., Sridangtip T., Sawedrungreang W., Krongtaew C. (2012). Utilization of Sugarcane Bagasses
for Lactic Acid Production by Acid Hydrolysis and Fermentation Using *Lactobacillus Sp*. Int. J. Chem. Mol.
Nucl. Mater. Metall. Eng..

[63] Outeiriño D., Costa-Trigo I., Ochogavias A., Pinheiro de Souza Oliveira R., Pérez Guerra N., Salgado J. M., Domínguez J. M. (2024). Biorefinery
of Brewery Spent Grain to Obtain Bioproducts with High Value-Added
in the Market. N. Biotechnol..

[64] Rivera O. M. P., Moldes A. B., Torrado A. M., Domínguez J. M. (2007). Lactic
Acid and Biosurfactants Production from Hydrolyzed Distilled Grape
Marc. Process Biochem..

[65] Wang C., Shan Y., Shen Y., Fu W., Li J., Blersch D., Wu W., Shi S., Han L. (2024). Detoxification
of Corn Stover Prehydrolysate by Different Biochars and Its Effect
on Lactic Acid Fermentation. RSC Adv..

[66] Bustos G., Moldes A. B., Cruz J. M., Domínguez J. M. (2005). Influence
of the Metabolism Pathway on Lactic Acid Production from Hemicellulosic
Trimming Vine Shoots Hydrolyzates Using *Lactobacillus Pentosus*. Biotechnol. Prog..

[67] Bustos G., Moldes A. B., Cruz J. M., Domínguez J. M. (2004). Production
of Fermentable Media from Vine-Trimming Wastes and Bioconversion into
Lactic Acid by *Lactobacillus Pentosus*. J. Sci. Food Agric..

