# Supplementary material for: Lactic Acid Production from Sugarcane Bagasse Hydrolysates by Lactiplantibacillus Strains
Source: ACS Omega. 2025 Oct 26;10(43):51891–9. doi: 10.1021/acsomega.5c08221 (PMC12593970; doi:10.1021/acsomega.5c08221)
Supplement: Supplementary file 1 [file ao5c08221_si_001.pdf]

Supporting information

LACTIC ACID PRODUCTION FROM SUGARCANE BAGASSE HYDROLYSATE BY *Lactiplantibacillus* STRAINS

*Michelle C. A. Xavier[1] *; Giancarlo S. Dias[2]; Saartje Hernalsteens[3]; Telma T. Franco[2].*

[1] Federal University of Tocantins (UFT), NS-15 Ave., Quadra 109 - Alcno 14, North, 77001-090, Palmas- Tocantins, Brazil.

[2] State University of Campinas (UNICAMP), 500, Albert Einstein Ave., 13083-852 Campinas - São Paulo, Brazil.

[3] Soochow University (SUDA), No.1 Shizi Street, Suzhou, Jiangsu 215031, China.

Corresponding Author:

Dra. Michelle C. A. Xavier, Email: michellecax@mail.uft.edu.br

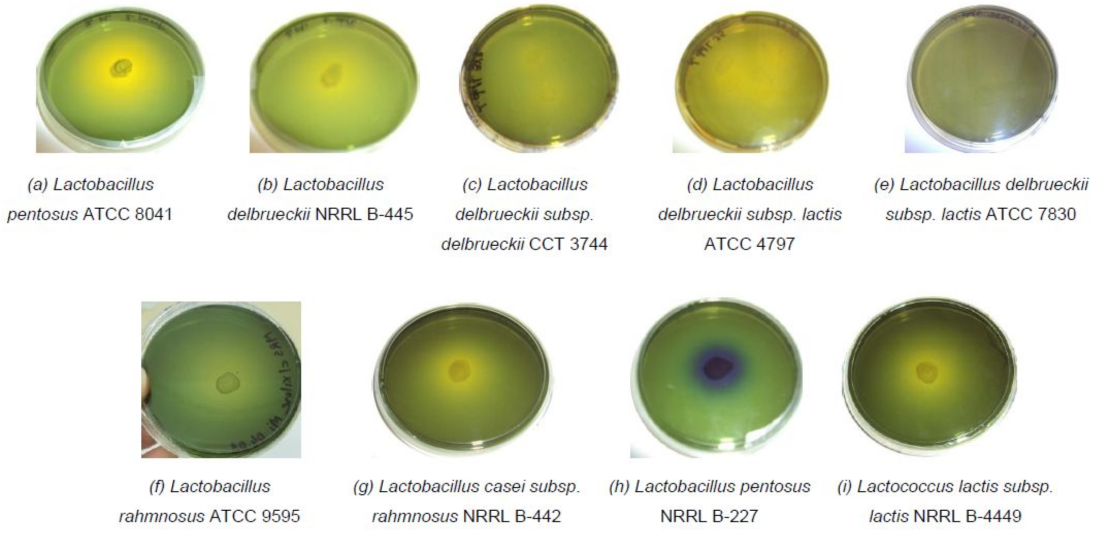

Figure S1. Acidification test in solid MRS_{xyl} medium using bromocresol green and bromocresol purple pH indicators for the *Lactiplantibacillus* strains.
